# Autophagy in human articular chondrocytes is cytoprotective following glucocorticoid stimulation

**DOI:** 10.3892/mmr.2014.2102

**Published:** 2014-04-01

**Authors:** NING LIU, WENBO WANG, ZHE ZHAO, TAO ZHANG, YUWEN SONG

**Affiliations:** Department of Orthopaedic Surgery, The First Affiliated Hospital of Harbin Medical University, Harbin, Heilongjiang 150001, P.R. China

**Keywords:** chondrocytes, dexamethasone, glucocorticoid, autophagy, apoptosis

## Abstract

For the past 60 years, glucocorticosteroid (GC) drugs, including prednisone and dexamethasone (Dex), have been used for the treatment of early stage osteoarthritis (OA). However, multiple administration of GCs may destroy the articular cartilage. It has been previously reported that GC treatment may also lead to the initiation of autophagy, which is an essential mechanism for cell homeostasis and survival. Rapamycin (Rapa), an inhibitor of the mammalian target of Rapamycin, may cause a degeneration-associated pathology in organs and induce autophagy in a variety of cell types, which has been applied in the treatment of experimental OA. A previous study by our group observed that GC apparently increases the apoptosis of chondrocytes, resulting in the inhibition of extracellular matrix synthesis. Therefore, the present study aimed to further examine the effects of autophagy in chondrocytes under GC treatment and to verify the molecular mechanisms involved in the cytoprotective role of Rapa. Short-term GC treatment did not significantly inhibit chondrocyte viability, while cell autophagy was increased. In addition, upregulation of autophagy by Rapa prevented the expression of apoptosis-associated genes and improved cell activity. In conclusion, the present study revealed that increased autophagy is an adaptive response to protect chondrocytes from short-term GC exposure, whereas prolonged GC treatment decreases autophagy and increases apoptosis *in vitro*. Upregulation of autophagy by Rapa may protect chondrocytes against the adverse effect induced by GC.

## Introduction

Osteoarthritis (OA) is the most prevalent type of joint disease in adults and is characterized by chronic pain and degradation of articular cartilage (1,2). Treatments for early stage OA initially focus on reducing pain and inflammation, and preventing the cartilage from further mechanical injury. Glucocorticosteroids (GCs) are intra-aritcular therapeutics first used in for anti-inflammatory treatment of rheumatoid arthritis in 1951 and are effective in pain relief and functional improvement (3–6). However, long-term and/or high-dose treatment with GCs may be harmful for articular cartilage metabolism and may induce apoptosis in chondrocytes by destroying the endoplasmic reticulum and mitochondria (7,8). In cartilage, chondrocytes are the sole resident cells that are involved in the synthesis of the extracellular matrix (ECM). Cartilage homeostasis requires sufficient ECM composition to exert its biomechanical functions. Therefore, inhibition of the growth of chondrocytes may reduce cartilage formation and possibly weaken the matrix structure.

Autophagy is characterized by the formation of an autophagosome. As the essential cellular homeostasis mechanism, the autophagosome may recycle the damaged and dysfunctional organelles and molecules (9,10). Therefore, autophagy is considered a protective process for cell survival under metabolic stress and adverse stimulus (11). Thus, impairment of autophagy may induce a variety of human diseases. Due to the relatively low rate of proliferation of chondrocytes, autophagy appears to be important in maintaining cell survival and biosynthetic function (12,13). However, whether the interaction between autophagy and apoptosis is involved in the effect of GC on normal human chondrocytes remains to be elucidated.

Rapamycin (Rapa) is a lipophilic macrolide antibiotic, which triggers autophagic functions by inhibiting the mammalian target of rapamycin (mTOR) (14). mTOR, a nutrient-sensing kinase which acts as a controller of ribosomal biogenesis and protein synthesis, may directly suppress cell autophagy (15). In addition, imbalances of this kinase are involved in metabolic diseases (16). Based on the potential of inducing autophagy, Rapa has been suggested to protect against certain degenerative conditions (17–19).

Recently, a study by our group demonstrated that short-term GC treatment did not suppress chondrocyte growth significantly, whereas long-term exposure to GCs induced apoptosis (20). Thus, the aim of the present study was to determine whether autophagy is one of the survival pathways through which chondrocytes respond to short-term GC treatment and whether pharmacological enhancement of autophagy by Rapa may serve as a protective mechanism against the adverse effects of long-term GC exposure.

## Materials and methods

### Chemicals and reagents

Dexamethasone (Dex), Rapa, monodansylcadaverine (MDC), Hoechst 33342 and dimethylsulfoxide (DMSO) were purchased from Sigma-Aldrich (St. Louis, MO, USA). A CCK-8 Cell Counting Kit was obtained from Beyotime Laboratories (Beyotime Institute of Biotechnology, Haimen, China). Annexin V-fluorescein isothiocyanate (FITC) and propidium iodide (PI) were purchased from BaoSai Biotechnology Co., Ltd. (Beijing, China). Antibodies against microtubule-associated protein light chain 3 (LC3), beclin-1, caspase-9, poly (adenosine diphosphate-ribose) polymerase (PARP), collagenase II, aggrecan and β-actin were purchased from Santa Cruz Biotechnology, Inc. (Santa Cruz, CA, USA).

### Human chondrocyte isolation and culture

Human articular cartilage tissue samples from the knee joint were obtained from 10 donors (average age, 50.2 years) undergoing surgery for tibial plateau fracture or total knee arthroplasty. These surgeries were approved by the ethics committee of The First Affiliated Hospital of Harbin Medical University (Harbin, China). The non weight-bearing area of the cartilage, without any abnormalities, was harvested and washed in sterilized saline. Next, the tissue was sliced into smaller sections of 1 mm^3^ and digested with trypsin and collagenase II for 5 h at 37°C. The mixture was filtered, centrifuged (0.2 × g) and washed in phosphate-buffered saline (PBS) at least three times for cell purification. Finally, the cells were transferred into a culture flask and incubated at 37°C in a 5% CO_2_ incubator with 10% fetal bovine serum in Dulbecco’s modified Eagle medium (DMEM) (Gibco-BRL, Carlsbad, CA, USA). Following the above procedure, the second-passage chondrocytes were used for further experiments.

### Cell viability assay

Chondrocytes (8×10^3^) were seeded into 96-well plates and cultured for 24 h. Based on the previous results, the cells were treated with 100 μM/l Dex in culture medium for the following times: 6, 12, 24, 48 or 72 h. Cells were treated with DMSO only as the untreated control (control group), and one group was pretreated with 10 μM/l Rapa for 2 h. At each time-point, 10 μl CCK-8 kit solution was added to each well and plates were incubated at 37°C for ~1 h. Next, a microplate reader (Thermo Fisher Scientific Inc., Walthom, MA, USA) was used to measure the optical density (OD) at 450 nm. The experiment was repeated at least five times. The survival rate of cells (%) = (experimental group OD value - blank group OD value)/(control group OD value - blank group OD value), and the inhibition rate of cell proliferation (%) equals to 100% minus the survival rate.

### Flow cytometric assessment of apoptosis

Following the respective treatment as above, the cells were washed with ice-cold PBS, harvested and counted. The cells (1×10^5^) were suspended in 500 μl binding buffer containing Hepes/NaOH (pH 7.4), NaCl and CaCl_2_ (BaoSai Biotechnology Co., Ltd.) and incubated with 10 μl Annexin V-FITC and 5 μl PI for 20 min. The rate of apoptosis (%) was measured by a flow cytometer (Epics Altra II; Beckman Coulter Inc., Brea, CA, USA). In addition, for Hoechst staining, the cells were seeded into 6-well plates and cultured for 24 h. After treatment with or without 100 μM of Dex for 72 h post-treatment, the cells were washed in cold PBS twice and were labeled with Hoechst 33258 staining solution (Beyotime Institute of Biotechnology), and maintained at 37°C in the dark for 20 min. The cells were then observed and imaged by fluorescence microscopy (Leica Microsystems, Wetzlar, Germany), with excitation at 350 nm and emission at 460 nm.

### MDC assay

Chondrocytes were seeded on sterile coverslips in tissue culture plates. Following treatment with Dex and/or Rapa for the indicated times, the cells were incubated with MDC (0.1 mM) for 30 min at 37°C. Following incubation, cells were washed three times with PBS and immediately visualized by fluorescence microscopy (Leica Microsystems). Excitation wavelengths were 360–380 nm and Olympus DP version software (Olympus Corporation, Tokyo, Japan) was used.

### Transmission electron microscopy (TEM) analysis

Chondrocytes were treated with Dex and/or for designated times as described for the MDC assay. Cells were then washed with 0.1 M cacodylate buffer (pH 7.4) and fixed with 2% glutaraldehyde in PBS for 24 h at 4°C. Samples were then processed following the standard procedure (21). A Zeiss transmission electron microscope (Carl Zeiss, Thornwood, NY, USA) was used to examine the section samples.

### Western blot analysis

Chondrocytes (2×10^6^) were pretreated with or without Rapa (10 μM/l) 2 h prior to Dex treatment. The cells were collected and western blot analysis was performed as previously described (19). Briefly, the cells were washed with ice-cold PBS and sonicated in radioimmunoprecipitation assay buffer and homogenized and cellular debris was removed by centrifugation (0.2 × g). The protein concentration was determined using the Bio-Rad DC protein assay (Bio-Rad Laboratories, Hercules, CA, USA). The protein lysates were separated by 12 or 15% SDS-PAGE and transferred to polyvinylidene difluoride membranes. The membranes were blocked with 5% bovine serum albumin (Beyotime Institute of Biotechnology) and then incubated with LC3 and Beclin-1 primary antibodies (1:500), Cleaved caspase-9 and PARP primary antibodies (1:1,000) overnight at 4°C and subsequently with horseradish peroxidase-conjugated anti-rabbit (1:1,500) or anti-mouse (1:2,000) secondary antibodies. The protein blots were visualized by enhanced chemiluminescence (Millipore, Billerica, MA, USA) with Chemilumino Analyzer LAS-3000 (Fujifilm, Tokyo, Japan). Quantity One 1-D Analysis software (Bio-Rad Laboratories) was used to analyze the band density of each blot. Anti β-actin was also used as an internal control and to confirm that each sample contained a similar quantity of protein.

### Statistical analysis

All the experimental data presented were confirmed in at least three independent experiments, unless otherwise indicated. The experimental results are expressed as the mean ± standard deviation. Collected data were statistically analyzed using a one-way analysis of variance and the Fisher’s least-significant difference test with SPSS 19.0 software (SPSS Inc., Chicago, IL, USA). P<0.05 was considered to indicate a statistically significant difference.

## Results

### Effect of GC treatment on the viability of human chondrocytes

**Figure 1:**
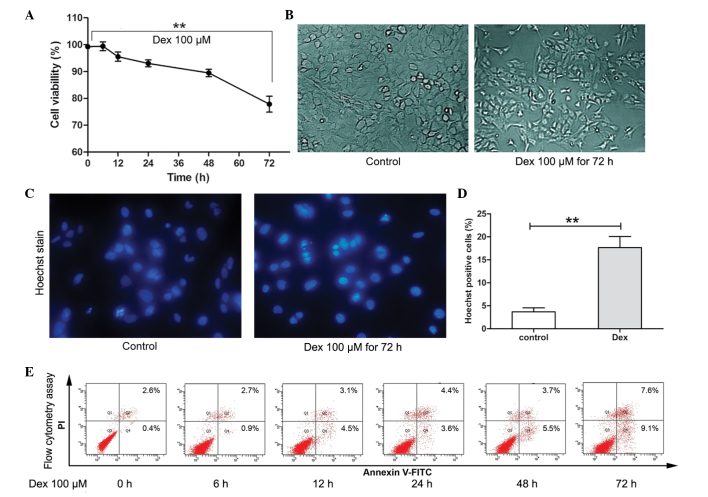
Inhibition rate and induction of apoptosis in human chondrocytes treated with Dex. (A) At different times of Dex treatment, the cell viability index (%) of six groups was assessed using the CCK-8 assay. The results are expressed as the mean ± SD and significant differences are indicated by asterisks (^**^P<0.01). (B) Morphological changes were observed by inverted microscopy (magnification, ×200). (C) Following Dex treatment for 72 h, apoptotic cells were stained with Hoechst 33342 and visualized by fluorescence microscopy (magnification, ×400). (D) Hoechst-positive cells (bright blue with nuclear fragmentation) are expressed as the mean ± SD and significant differences are indicated by asterisks (^**^P<0.01, vs. control). (E) Percentage of apoptotic cells following Dex treatment for different periods of time. The apoptotic index was determined by flow cytometry following Annexin V-FITC and PI staining. Dex, dexamethasone; FITC, fluorescein isothiocyanate; SD, standard deviation; PI, propidium iodide.

It has been previously observed that, following 72 h exposure, Dex at 100 μM/l suppressed cell growth significantly (18). Therefore, in the present study, the short-term effect of Dex stimulation on the viability of chondrocytes was determined using the CCK-8 assay. As shown in Fig. 1A and B, during short-term treatment (6–24 h), the average viability of chondrocytes remained >90%. However, following 72 h treatment, the viability of cells was decreased significantly (P<0.01) and cell morphological changes were observed. To further determine the cytotoxicity of Dex on chondrocytes, flow cytometry and Hoechst staining were used to assess apoptosis. As shown in Fig. 1C–E, following 100 μM/l Dex treatment for 6, 12, 24 and 48 h, a slight increase in the rate of apoptosis was detected (3.6–9.2%). However, following treatment for 72 h, the rate of apoptosis increased to 16.7%. Furthermore, Hoechst 33342 staining showed that cells treated with Dex for 72 h exhibited an increased ratio of Hoechst-labeled (bright blue nuclei indicative of apoptosis) cells compared with untreated cells (^**^P<0.01). These results indicated that short-term Dex treatment did not induce significant levels of apoptosis-associated cell death in chondrocytes; however, long-term treatment may induce apoptosis significantly.

### Short-term GC treatment induces autophagy in chondrocytes

**Figure 2:**
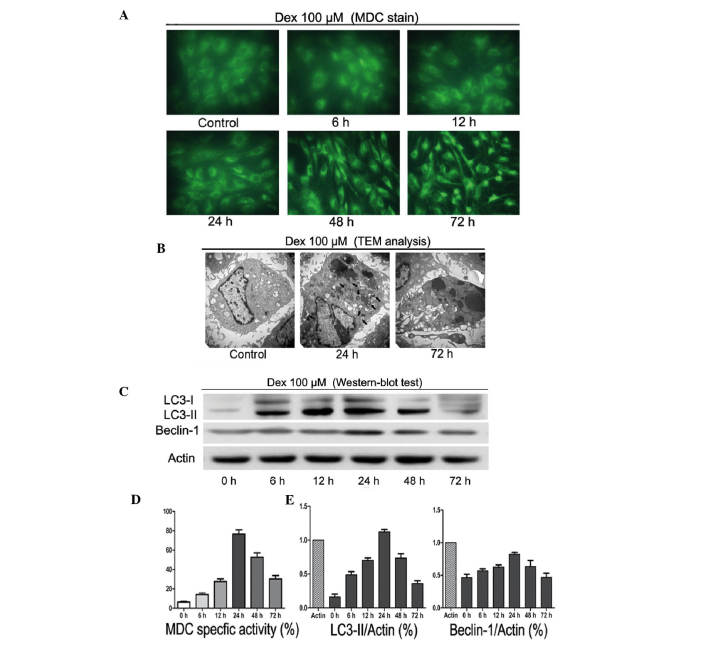
State of autophagy in human chondrocytes following Dex treatment. (A) MDC-labeled vacuoles were examined by fluorescence microscopy (magnification, ×400). (B) Formation of autophagic vacuoles were observed by TEM (magnification, ×15,000). Autophagic vacuoles are indicated by black arrows. (C) Western blot analysis of LC3-I, LC3-II and beclin-1 following incubation with Dex for different times. β-actin was used as a internal control. (D) The specific activity index (%) was obtained by flow cytometry. (E) Levels of proteins were normalized with respect to the β-actin band density using Bio-Rad Quantity One software. Data are expressed as the mean ± standard deviation. Dex, dexamethasone; MDC, monodansylcadaverine; TEM, transmission electron microscopy; LC3, microtubule-associated protein light chain 3.

To investigate the autophagic activity following Dex treatment, MDC staining, TEM and western blot analysis were used. As shown in Fig. 2A and D, 100 μM/l Dex induced autophagy in a time-dependent manner. Between 6 and 24 h of treatment, MDC-labeled vacuoles were significantly increased. By contrast, following 48 and 72 h Dex treatment, a decrease in the occurrence of cell morphological changes and MDC-labeled vacuoles was observed. TEM scanning showed a large number of free membrane structures and double-membrane vacuoles, which contained portions of cytosol and organelles, in the cytoplasm following treatment with Dex for 24 h as compared with untreated cells. These structures resembled pre-autophagosomal structures (PAS) or autophagosomes (Fig. 2B, black arrows scale bars). Western blot analysis showed that LC3-II and Beclin-1 protein levels significantly increased between 6 and 24 h of treatment (Fig. 2C and E). However, this trend was reversed following long-term exposure to Dex. These observations suggested that short-term treatment with GCs may induce autophagy in chondrocytes.

### Effects of activation of autophagy by Rapa on apoptosis of chondrocytes

**Figure 3:**
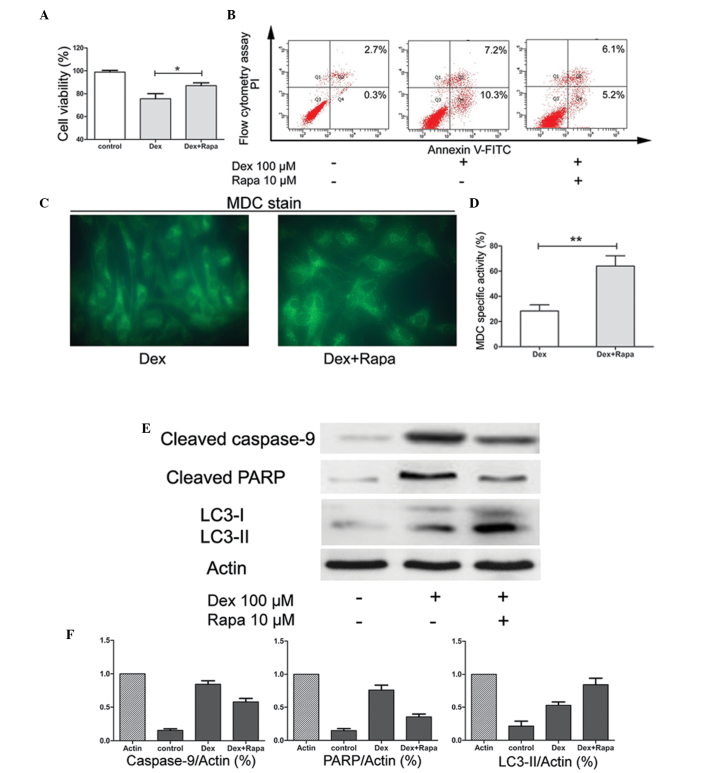
Effects of activation of autophagy by Rapa. (A) Chondrocytes were pretreated with or without Rapa and exposed to Dex for 72 h. The cell viability index (%) was obtained using a CCK-8 assay. Data are expressed as the mean ± SD and significant differences are indicated by asterisks (^*^P<0.05, vs. Dex). (B) Following treatment, the apoptotic index was determined by flow cytometry following Annexin V-FITC and PI staining. (C) Following Dex only or pretreatment with Rapa with subsequent Dex exposure for 72 h, the MDC-labeled vacuoles in chondrocytes were examined by fluorescence microscopy (magnification, ×400). (D) The specific activity index (%) was obtained by flow cytometry. Data are expressed as the mean ± SD and significant differences are indicated by asterisks (^**^P<0.01, vs. Dex). (E) Western blot analysis for cleaved caspase-9, cleaved PARP, LC3-I and LC3-II protein following 72 h of incubation of untreated samples, Dex-treated samples and samples pretreated with Rapa followed by Dex treatment. β-actin was used as a internal control. (F) Levels of proteins were normalized to the β-actin band density using Bio-Rad Quantity One software. Data were expressed as the mean ± SD. Rapa, rapamycin; Dex, dexamethasone; MDC, monodansylcadaverine; FITC, fluorescein isothiocyanate; SD, standard deviation; PI, propidium iodide; LC3, microtubule-associated protein light chain 3; PARP, poly (adenosine diphosphate-ribose) polymerase.

It has been reported that Rapa may induce autophagy in chondrocytes. To further confirm the protective effect of Rapa in chondrocyte exposure to GC, the cells were pretreated with Rapa 2 h prior to treatment with Dex. The results suggested that, compared with Dex only treatment, pre-culture of the cells with Rapa enhanced the cell viability significantly (P<0.05; Fig. 3A) and reduced the apoptotic rates (Fig. 3B). Furthermore, the activity of autophagy was stimulated by Rapa. The number of MDC-specific vacuoles was significantly increased (P<0.01; Fig. 3C and D). Finally, LC3-II protein expression was also upregulated, whereas the expression of apoptosis-associated gene caspase-9 and the protein PARP were downregulated (Fig. 3E and F). These results suggested that the apoptosis induced by Dex may be through the activation of the caspase signaling pathway and that Rapa may maintain chondrocyte survival in the presence of GC by enhancing autophagy and suppressing apoptosis.

## Discussion

OA is the most common type of joint disease worldwide, which is mainly characterized by chronic, irreversible degradation and erosion of cartilage (1,22). For the majority of patients, joint pain and movement disorders are the primary clinical symptoms. At early stages, if the cartilage damage is not serious and joint pain is the major complaint, nonoperative therapy is preferred by the majority of clinicians (23). Glucocorticoid drugs, including Dex and prednisone, are effective anti-inflammatory agents in intra-articular injection therapy. Numerous studies have demonstrated the positive effects of GC in the treatment of early OA (24,25). However, GC treatment alters cartilage metabolism and changes the intra-articular environment (7,8,26). Therefore, a number of studies have hypothesized that the repeated use of GC may facilitate cartilage degeneration (27,28). In a recent study, long-term GC treatment was observed to inhibit the growth of chondrocytes and restrain the synthesis of the extracellular matrix (20). In the present study, it was observed that short-term GC treatment did not significantly reduce chondrocyte viability.

Autophagy, or the autophagic process, is a well-conserved mechanism among species and has been confirmed to be important in various biological events (9,10,11,28). This system is characterized by the formation of double-membrane autophagic vesicles, which contain cytoplasm, and/or organelle degradation by lysosomes for material recycling and ATP generation (29). Among the human autophagy genes, Beclin-1 and LC3 are major regulators and markers of the autophagy pathway (30). Beclin-1 forms a complex with type III phosphatidylinositol that allows nucleation of the autophagic vesicles. During the process of autophagy activation, LC3-I is converted into LC3-II, which is then attached to the membrane of the autophagosome.

Chondrocytes are involved in maintaining a balance between synthesis and degradation of the ECM. Therefore, cell death is associated with the breakdown of cartilage (31–33). In 2004, the term ‘chondroptosis’ was the first to define this type cell death, which includes classical apoptosis and autophagy (34). In a number of instances, the cell switches between the above two responses in a mutually exclusive manner (35). As for the non-apoptotic mechanism, numerous studies have examined the roles of autophagy in normal cartilage metabolism and pathological conditions. Normal chondrocytes have a certain degree of autophagy and the aging-associated loss is linked with cell death (12). By contrast, the decreased autophagy with age may be an explanation for OA and activation of autophagy may reduce the severity of experimental OA (36). Therefore, in the early stages of OA, autophagy may be an adaptive response to avoid chondrocyte death (37). However, in deep zones of OA cartilage, due to the abnormal subchondral bone ossification, apoptosis is the only death process observed (37). In the present study, an increase in autophagic activity induced by short-term GC treatment was detected. The results show that only 6 h of chondrocyte exposure to Dex caused an upregulation in cell autophagy. This trend reached a maximum following 24 h of treatment. Continuous exposure to Dex led to the downregulation of autophagic activity. These resuls were obtained by MDC staining, which identified these trends in the autophagic activity of cells. The above results suggest that short-term treatment with GCs may also activate autophagy, whereas long-term exposure to GCs increases apoptosis. Autophagy is likely to be a self-protective process in chondrocytes in response to stimulation with GC.

To further determine the role of pharmacologically enhanced autophagy associated with the effects of Dex on chondrocyte viability, Rapa was employed as an autophagy inducer. The results suggested that, compared with Dex only treatment, Rapa enhances the viability of the cells after 72 h. Next, western blot analysis confirmed that the pathological apoptosis markers caspase-9 and PARP were downregulated by Rapa, while the autophagy marker LC3 was upregulated. Rapa is a specific inhibitor of mTOR kinase, which regulates autophagy activation (18). mTOR is a nutrient-sensing kinase that integrates input from the energy status of the cell. Therefore, this kinase controls the ribosomal biogenesis and protein synthesis (16,38). It has been demonstrated that this kinase is also involved in the cell death of chondrocytes in OA. Firstly, the activation of mTOR may suppress chondrocyte proliferation and differentiation by phosphorylation of the ribosomal protein S6 (39). Secondly, the inhibition of autophagy by mTOR caused OA-like changes in gene expression (40). Furthermore, mTOR inhibition may suppress the expression of A disintegrin and metalloproteinase with thrombospondin motifs 5 and interleukin (IL)-1β (36). Induction of proteinase expression in chondrocytes by IL-1β was reactive oxygen species (ROS)-dependent. Furthermore, increased ROS activity has been suggested to inhibit the growth factor expression and enhance the production of matrix metalloproteinases (41). However, the activation of autophagy by Rapa markedly reduces the ROS levels induced by IL-1β and reduces the severity of experimental OA (35,36,42,43). GCs function as an anti-inflammatory agent. Therefore, in the present study, it was hypothesized that the apoptosis of chondrocytes may not be associated with the toxicity of inflammatory factors, but with the metabolic stress caused by GCs. Thus, pharmacologically enhanced autophagy by Rapa may act as an adaptive response to protect the chondrocytes from a relatively long-term exposure to GCs.

In conclusion, in regard to chondrocytes, the self-activation of autophagy is a protective mechanism against apoptosis under short-term treatment with GCs. However, persistent exposure to GCs may cause the cell to become dysfunctional, resulting in apoptosis. Furthermore, pharmacologically enhanced autophagy by Rapa provided novel insights into the treatment of the adverse effects of GCs on cartilage. Further study is required to elucidate the interaction between ECM synthesis and autophagy in chondrocytes.
